# Towards the revival of oscillation from complete cessation in stochastic systems for application in molecular biology

**DOI:** 10.6026/97320630016274

**Published:** 2020-03-31

**Authors:** Shakti Nath Singh, Md.Zubbair Malik, RK Brojen Singh

**Affiliations:** 1 School of Computational and Integrative Sciences, Jawaharlal Nehru University, New Delhi 110067, India

**Keywords:** Stochastic, Time delay, noise, oscillation, amplitude

## Abstract

Delay and noise are inevitable in complex systems that are common in biochemical networks. The system is often disturbed at various states irrespective of the size (small or large) of delay and noise. Therefore, it is of interest to describe the significance of delay and noise in stochastic Willamowski-Rossler chemical oscillator model using a delay stochastic (having random probability distribution) simulation algorithm. Oscillating dynamics moves to stable fixed point when delay at a fixed magnitude of noise drives the system from oscillating state to stochastic amplitude death state (complete cessation). However, the amplitude death state is induced to a revived oscillating state in stochastic system (which is far from equilibrium state) for noise with a fixed value of delay. Thus, significantly large and small noise induces the dynamics of the system to amplitude death state. Hence, we describe the interplay of delay and noise in stochastic systems for the proper and efficient functioning of the complex system that are frequent in biological networks.

## Background

Functional organization and regulation in biochemical systems are the outcome of chain of molecular interactions/events defined by sets of well-defined reactions in various pathways. These molecular events in such systems occur in a certain random manner and needs to solve these sets of reactions to understand systems' behaviour [1]. Such complicated reaction sets are generally solved by using stochastic simulation techniques because of difficulty in solving them analytically [2]. Despite each reaction has their own role in a system, some reactions are fast and some are slow in nature, and overall interaction exhibit emergent behaviour which are generally inherent properties of the system [3]. Dynamics of biological systems are a continuous set of actions among molecules triggered by chemical reactions that leads to certain task or function in the cell [2]. The dynamics of such system involves the properties of the system and can explain well state evolution of the systems with time, which may correspond to various important cellular states [2]. Because of small population of molecular species participation in reaction channels of biological systems with random interactions among them (random collisions among molecular species and random firing of reactions) exhibit randomness in the system [1]. These randomness and fluctuations become significant in case of very low molecular species population in the biological systems and then to explain the dynamics of such systems we need to deal the mathematical models by considering the noise with stochastic modelling approach [1,2].

Gillespie considered and modelled that all reactions finish instantly as they start and the process is Markovian [1,2]. The stochastic simulation algorithm (SSA) or Gillespie algorithm was based on two random events in reaction time and choice of reaction, and noise in the dynamics become an inherent property of the complex system [2]. The algorithm did not considered delay into account. However, various experimental evidences validate the existence of delayed reactions in biological systems, for example, transcription and translation reactions in gene expression process [4-6], protein degradation, feed backing in biological systems [7] and gene inhibition mechanism [8] etc. In one of the works of Duan et al., they studied the dynamics of calcium of cells in terms of anti synchronization as effect of the short, moderate, and long-time delay in the system [9]. Hence, one needs to incorporate time delay in such systems to capture accurate dynamics of the system. Such process become non-Markovian, and SSA was generalized by considering delay [10,11].

Delay has various roles in regulating dynamical systems. In deterministic systems, it has been found two contrast roles delay, namely, delay induced oscillation death [12] and amplitude death [13] on one hand, and on the other hand, revival of oscillation driven by delay [14]. There have been few reports that delay can induce oscillations in stochastic systems, namely, exhibiting oscillations in p53 regulatory network [15, 16], observation of on and off states of genes in toggle switch [17], emergence of delay induced stochastic oscillations in gene regulation [10] etc. Further, in some studies in Drosophila, Neurospora and other organisms showed that the oscillations in their dynamics established during the transcriptional regulations induced by delay [18-21]. Experiments, then, has proven that these oscillations are caused by induced delay in gene regulation networks [22,23]. Those are few reports that delay can induce different synchronization for different forms of oscillatory behaviours (quasi-periodic, chaotic oscillations etc)[9]. Further, delay can also cause various forms of behaviour in the system dynamics, and even disappearance of coherence [37]. Biologically, if any system parameter (may be delay, noise etc) induce switch off of the oscillation, the state may correspond to the inactive state or system failure which can be correlated with cell death or apoptosis. However, the revival of oscillation technique can play an important role to cure or prevent such failures. However, the role of delay by intertwining with noise to amplitude death, switching mechanism and in revival of oscillations in stochastic systems is not fully studied. We studied the switching mechanisms/states of oscillations and their relationships with delay time using the three variables chemical oscillator model proposed by Willamowski and Rossler [24,27].

## Methods:

### Theoretical framework of delay stochastic simulation algorithm

Biochemical reactions in a system can be broadly divided into two types: delayed and non-delayed reactions. Let us take a system with N chemical reactions R _i_:i = 1,2,...,N in which M number of chemical species S ={S_1_,...,S_M_} take part in reaction channels. State change dynamics of chemical species are stored in state vector X(t)=[ X_1_,X_2_,X_3_,...,X_n_]^T^, where for n^th^ species population at time t would be X_n_ according to this state vector X(t). Let us assume there are N_d_ numbers of delayed and N_n_ numbers of non-delayed reactions in a system where total number of chemical reactions N = N_d_ + N_n_ .Time delays in the system (delayed reactions) are τ_i_ = {τ_1_,τ_2_,...,τ_n_} in the probability space with the consideration (τ_i_ - τ_i - 1_)∼ f(τ), then the time evolution of the configurational probability P(X,t) of the system can be described by then the modified master equation which incorporates delay reactions, known as delay stochastic master equation (DSME) [24] is given by,

(∂/∂t P(X,t)= -∑_(X)_W^[n]^_ X'→ X_P(X,t)+∑_(X')_ W^[n]^_X→X'_ P(X',t) 

-∑_(X)_∑_(∀X_i_∈X)_ W^[d]^_(X'→X)_ P(X,t;X_i_,t-τ_i_) 

-∑_(X')_∑ _(∀X_i_∈X')_ W ^[d]^_(X→X')_ P(X,t;X_i_ µ_i_,t-τ_i_)

→ (1)

The probability of being the system in state X at time t is the function P(X,t). Where, µ_i_ is state change or the stoichiometric ratio. Transition probabilities for delayed reactions {W^[d]^} and transition probabilities for non delayed reactions {W^[n]^}. The first two terms of the R.H.S. of equation (1) are non-delay parts, and the second two terms are for delay part. Such delay stochastic master equation (1) for complex system is quite difficult to solve, and hence needed to use computational techniques to solve it. The delay reactions generally follow non-Markovian processes [10], and their time evolution trajectories cannot be simulated using SSA. In order to simulate stochastic systems containing delayed reactions, one must use delayed stochastic simulation algorithm (DSSA) proposed by Bratsun et al. [10] and Barrio et al.[10].In this simulation process, whenever the delayed reactions occur there will be a change in population of chemical species and the corresponding propensity function will be changed with every time step encountering delay reactions. This DSSA is the extension of original Gillespie algorithm by including delay reactions [2].The pseudo-code of the DSSA is given by,

### Pseudo-Code of DSSA

1.Initialize the population of molecular species {X} at time t = t_0_.

2.Calculate propensity functions {a} for all the reactions and a_0_=∑_(i=1)_^M^a_i_.

3.Generate two uniform random numbers r1 ,r2 between (0,1).

4.Calculate reaction time: τ=(1/a_0_)ln(1/r_1_).|

5.Firing of j^th^, j ∈ (1,...,M) reaction is determined by: ∑^(j-1)^_(i=1)_a_i_ <r_2_ a_0_ <∑^j^_(i=1)_a_j_

if {delayed reaction is scheduled in [t,t+τ'd) with τ'd < τ } then

 Update t←τ+τ'd, where τ'd is the first delayed reaction finishing time.

 Update state vector {X}:X = X + ∨_d_

 Go to Step 2.

 else

 if {non-delayed reaction to finish in this time interval [t,t+τ + κ_j_)} then 

 Update state vector {X}: X = X + ∨_j_

 Update t ← t + τ.

 Go to Step-2

 end if

 end if

 Stop 

**Figure 1:**
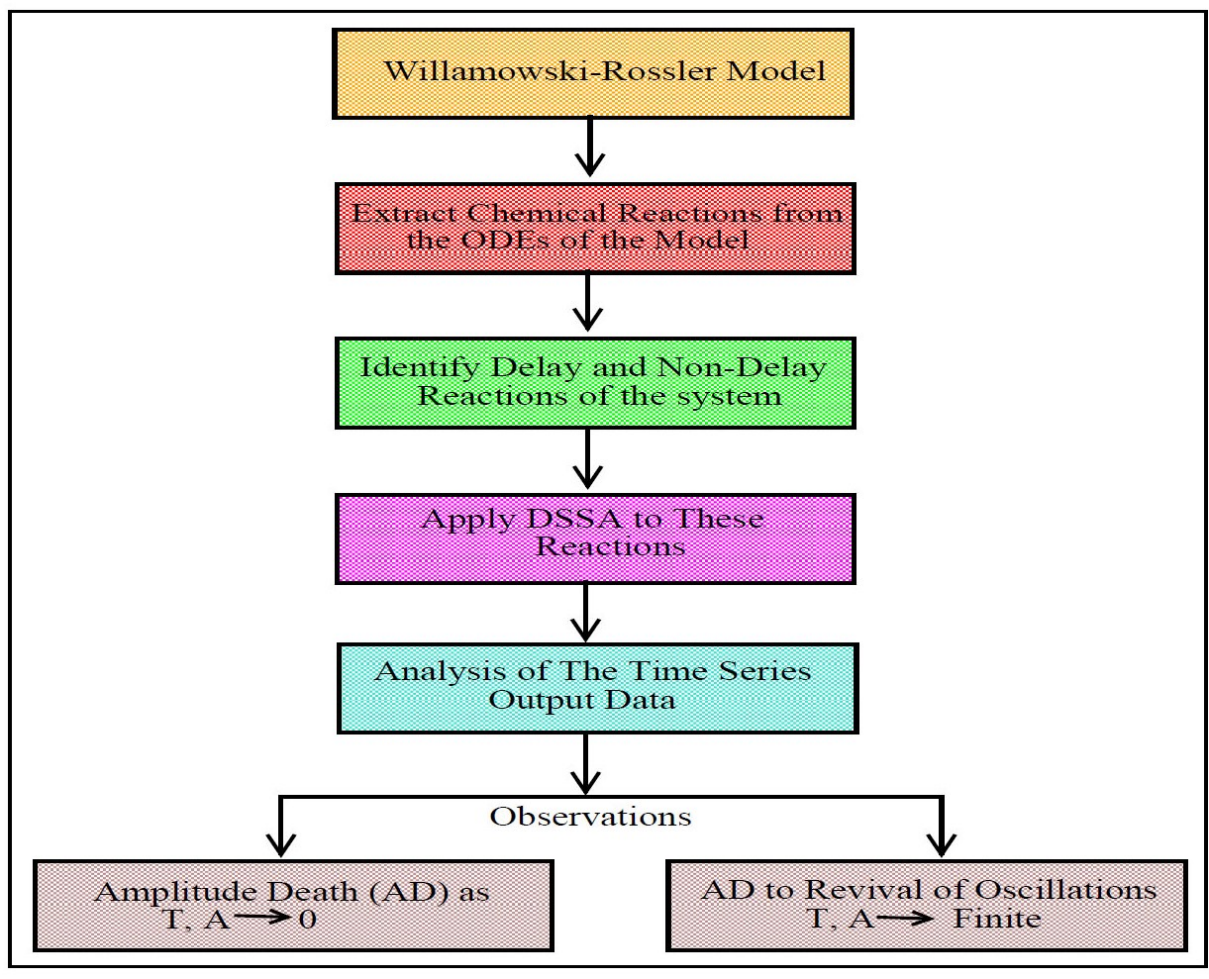
Flow chart of the methodology.

Iterate this algorithm until finishing time "t" for "M" numbers of reactions. The difference between Gillespie's SSA and the DSSA is handling the delayed reactions, remaining concepts are same. Whenever delayed reactions occur we store the finishing time τ_d_^'^ in a list, and will wait till that finishing time to update the state vector for molecular species affected by those delayed reactions. Once the simulation time reach to that finishing time of delayed reaction, the state vector "X" is updated accordingly, as well as update the simulation time (t+τ) with (τ_d_) as new time. Due to these changes in state vector of the molecular species, algorithm needs to recalculate the propensity functions and τ and "τ_d_" for next iteration. Further, the total numbers of the re-updating time "t" is exactly equal the total number of occurrences of the delayed reactions [26]. We have presented the flow chart of our methodology in figure (Figure 1) from initial data preparation steps to final time series data analysis steps.

### Stochastic chemical oscillator: Willamowski-Rössler model

We considered a well-known Willamowski-Rössler model for our study [24,27].This three-dimensional model (involves three variables) can be represented by a set of forward and reverse reactions, which can able to exhibit chaotic behaviour [27].The model reactions in the well-stirred system are given by,

U_1_+X⇔_(k_2_)_^(k_1_)^2X;X+Y⇔_(k_4_)_^(k_3_)^2Y;U_3_+ Y⇔_(k_6_)_^(k_5_)^ U_2_;X+Z⇔_(k_8_)_^(k_7_)^U_4_;U_5_+ Z⇔_(k_10_)_^(k_9_)^2Z →(2)

Where, {k_i_}; i = 1,2,...,10 are rate constants of the reactions in the model. {U_i_}; i = 1,2,...,5 are the equilibrium values the respective molecular species which are taken to be constants. We used this model for our study.

## Results and discussion:

We used DSSA to simulate Willamowski-Rossler model (2) by taking the parameter values: k_1_= 31.2,k_2_=0.2,k_3_=1.3,k_4_=0.1,k_5_=10.8, k_6_=0.12,k_7_=1.02,k_8_= 0.01,k_9_=16.5 and k_10_=0.5.The initial values of X,Y and Z are 20,20 and 20 respectively. We considered reactions 2Y→^k_4_)^X + Y and 2Z→^k_4_)^ Z+ U_5_ as the delay reactions and the rest eight reactions are taken to be non-delay reactions for our simulation and study. The choice of the delay reaction/reactions can be done as per the values of rate constant.

## Delay induced amplitude death:

**Figure 2:**
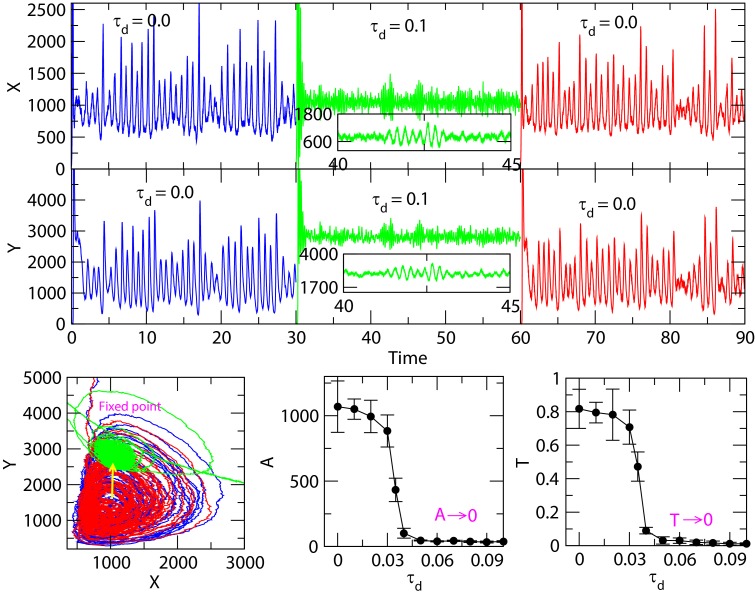
Delay stochastic simulation of the Willamowski-Rossler model: Initial population of X=20,Y=20,Z=20, simulated for time [0-90]. (a)Population of X with respect to time at time delay τ_d_ = 0.0 (blue) for time range 0-30,τ_d_ = 0.1 (green) for time range 30-60 and τ_d_ = 0.0 (red) for time range 60-90. (b)Population of Y with respect to time at time delay τ_d_ = 0.0 (blue) for time range 0-30, τ = 0.1 (green) for time range 30-60 and τ_d_ = 0.0 (red) for time range 60-90 (c) Population of Y versus population of X (d) Amplitude (A) of X (e) time period (T) of X as a function of τ_d_, where, points are means of the amplitudes with error bars.

We present the simulation results of the dynamics of X and Y for time t = [0 - 90], where, for ranges t = [0 - 30] and t = [60 - 90] are for delay time τ_d_ = 0, and delay with value τ_d_ = 0.1 is switched on for the range t = [30 - 60]. The system size is fixed at V = 0.1 during this simulation (Figure 2 upper two rows). The results show that for non-delay case (τ_d_ = 0), the X and Y dynamics show oscillatory behaviour with well-defined amplitude (A) and time period (T) with stochastic fluctuations in the dynamics (Figure 2 middle and right panel of the lowest row). Hence, stochastic oscillation is obtained for significantly small τ_d_→[0 - 0.02], where, A→1000±150, and T→0.77 ± 0.2. However, once the delay is switched on, the amplitudes and time periods of the X and Y dynamics are decreased monotonically, the scenario, which can be known as stochastic amplitude death. In stochastic amplitude death condition, A→0; T→0 for τ_d_ > 0.05, and oscillation dynamics moves to near fixed-point condition (Figure 2 left panel of the lowest row). In this case, oscillatory dynamics did not exactly, but nearly go off. This scenario is quite different from the earlier delay induced amplitude death observed in deterministic case of coupled system with delay [27] and distributed delay [28]. In our case, the delay in the system is in fact intrinsic, and is due to the intrinsic nature of some of the reactions involved in the system. The results indicate that delay is multi-functional, τ_d_=F(ξ,X,Y,Z), is sensitive to noise ξ, and has the capability of stabilizing as well as activating the system. This amplitude death in the dynamical system can be correlated to the system's failure leading to apoptosis in cellular dynamics.

## Noise induced revival of oscillation:

**Figure 3:**
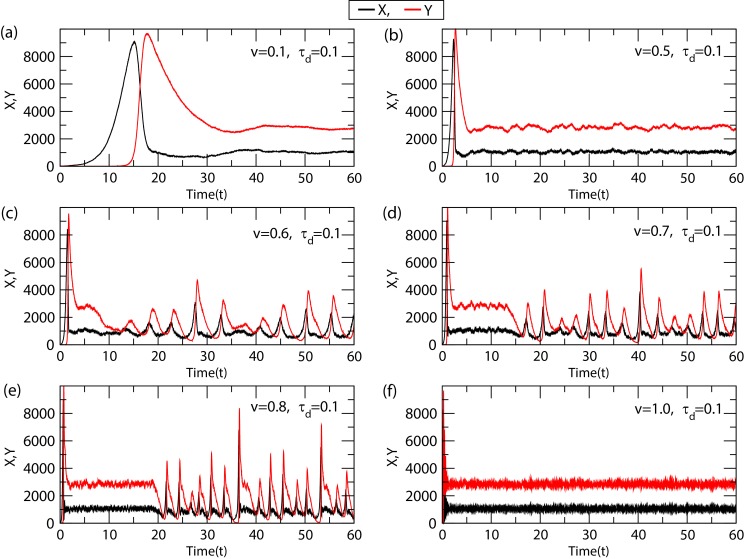
Noise induced system's dynamics: The time delay is fixed at τ_d_ =0.1 throughout the simulation. Dynamics of X and Y with respect to time, (a) for V = 0.1,τ_d_ = 0.1 (amplitude death case),(b) for V = 0.5,τ_d_ = 0.1(amplitude death case),(c) for V = 0.6,τ_d_ = 0.1 (revival of oscillation case), (d) for V = 0.7,τ_d_ = 0.1 (revival of oscillation case),(e) for V = 0.8,τ_d_ = 0.1 (revival of oscillation case), and (f) for V = 1.0,τ_d_ = 0.1 (amplitude death case).

We now consider the Willamowski-Rossler system (2) for stochastic amplitude death case V = 0.1,τ_d_=0.1 as obtained in the previous section (Figure 3a). The noise ξ associated with the system's dynamics can be characterized by system's size V by the relation ξ ∝ 1/√V [30]. Now, keeping delay fixed (τ_d_ = 0.1), we varied the system size V→[0.1-1.0] and studied the system's dynamics. We found that for a significantly large V (V = 0.6,0.7,0.8), the oscillations in the system became revived with well defined amplitude and time period (Figure 3 panels c,d and e). This scenario can be termed as stochastic revival of oscillations with the condition: A→f inite > 0, T→f inite > 0. Further increase in V (V = 1), the amplitude and time period of oscillations in the system became death: A,T → 0 (Figure 3 panel f). From these results we found that for a fixed delay, there is a certain range of V where the system's dynamics recover oscillations, beyond this range the system's dynamics moves to amplitude death scenario. In biological systems, keeping oscillations in the population dynamics of the participating molecular species is important for active and proper cell functioning and signal processing [31,32].

**Figure 4:**
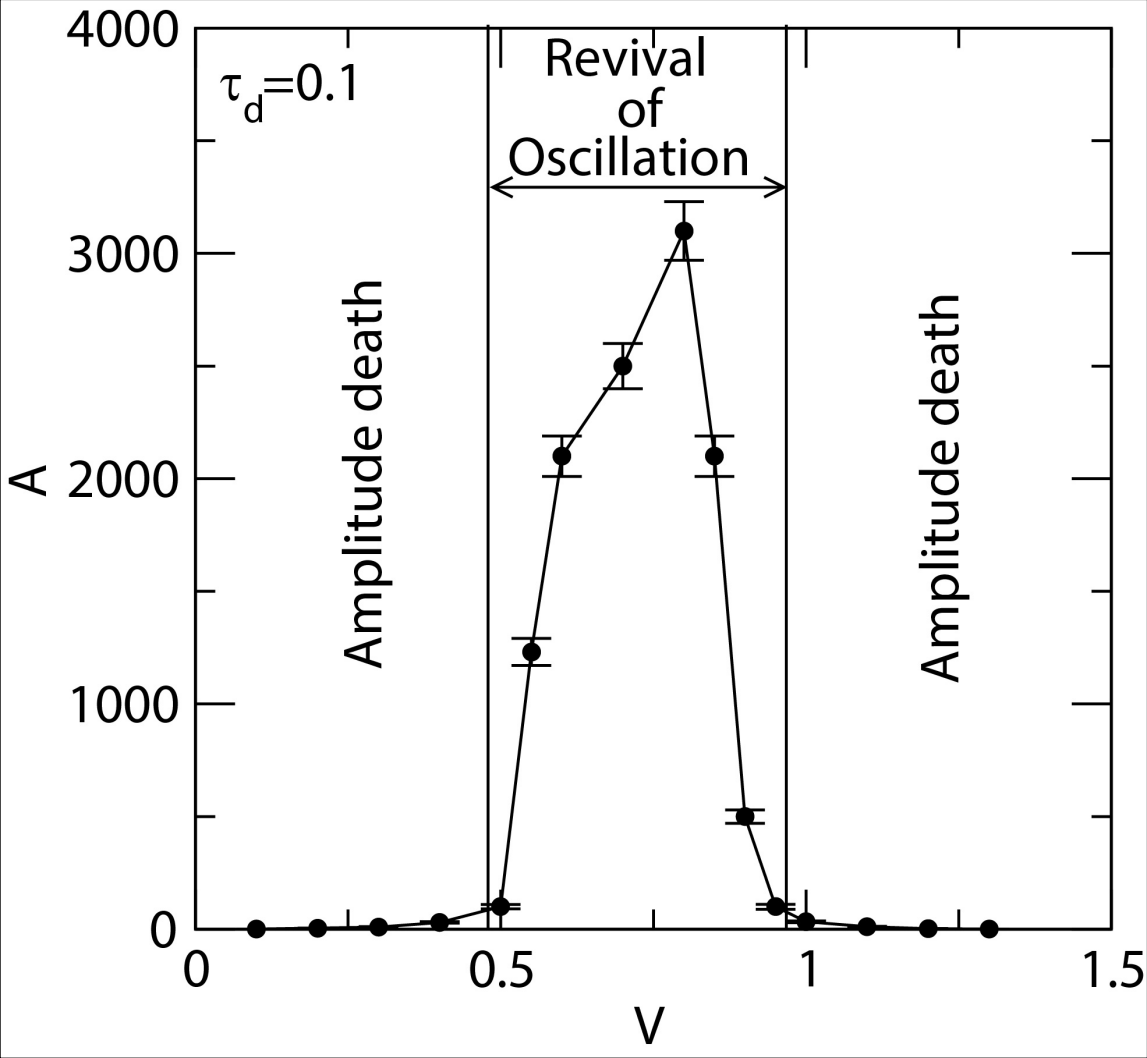
Dynamical phase diagram: Plot between amplitude of X as a function of V. Points are mean amplitudes between t = [20 - 60] with standard error bars.

The interplay of noise and delay is quite important and significant in maintaining a dynamical system active for efficient signal processing and functioning. We observed this phenomenon in the dynamical phase diagram in Figure 4 which showed that there is a certain range of V → [0.47 - 0.88] within which oscillations in the system's dynamics is well defined, A,T → f inite » 0, otherwise, the system collapsed to amplitude death, A,T → 0. This indicates that significantly small and large system's size V induce amplitude death indicating the system is either in normal or apoptosis state. During the revived oscillation state, which is in active state in general, the system is in non-equilibrium state. Hence, the noise is multifunctional and dependent on τ_d_,such that, ξ = G(τ_d_ ,V,X,Y,Z). Since noise and delay time are inherent parameters of biological systems, these parameters generally play important roles to regulate the system as well as protect the system mechanisms against various forms of external attacks and internal system failures.

## Conclusions:

Delay and noise are two important inherent parameters, which involved with system's dynamics. Their individual roles as well as interplay between them in regulating any dynamical system are quite important to investigate to predict various dynamical states evolved with the system. To understand these roles, we study Willamowski-Rossler model by dividing the reactions into two groups, delay reactions where, delay is involved in the reactions, and rest of the reactions non-delay reactions, where, delay is not involved in these reactions. Then we used DSSA to simulate the dynamics of the system to explore the roles of delay, noise and their interplay to regulate the system. For fixed noise, we observed that delay could induce stochastic amplitude death in the system. In biological systems, this state may correspond to either normal or apoptotic state [32]. The signature of amplitude death in system's dynamics can be used in various problems, for example, in hearing mechanisms in frog [33] etc. Hence, in biological systems, such significant changes in the rhythmic activity could lead to various pathogenic/disease states [34]. Further, delay is quite sensitive in regulating the system, and could able to activate and stabilize the system.

Noise has very interesting role in regulating Willamowski-Rossler dynamical system. For fixed delay, which keep the system at amplitude death regime, if we decrease the intrinsic noise strength (increasing system's size V) we observed that the suppressed oscillation due to amplitude death become revived with well defined amplitude and time period for a certain range of noise magnitude. If we further decrease the magnitude of noise, the system dynamics moved back to amplitude death again. The revival of oscillations for a certain range of noise magnitude in stochastic system is quite interesting which may open up many other significant roles of noise and delay. Because this state of revival of oscillation can be thought of bringing the collapsed state to revive active state, where, the system is far from equilibrium and works comfortably with efficient signal processing. Hence, the role of noise in this regime is quite constructive in this regime where stochastic resonance [36] can takes place. Further, it can also be understood that nature provides delay and noise as system's inherent properties to enable to work efficiently subjected to any internal and external perturbations. Study of interplay of delay and noise in field of biological systems, especially in neuroscience, cognitive science and complex systems could be quite interesting. Further, the exhibited dynamical states (amplitude death, reviving oscillating states, forms of oscillating behaviour etc) and the degree of changes in the behaviour of these states can be used as the indicators of conditions of patients under various diseases. Explore of these states in the dynamical patients' data is quite challenging and important which can be beneficial to clinical trials and medical practitioners.
